# Knowledge of hepatitis C and awareness of reinfection risk among people who successfully completed direct acting antiviral therapy

**DOI:** 10.1371/journal.pone.0265811

**Published:** 2022-03-23

**Authors:** Kiana Yazdani, Katerina Dolguikh, Wendy Zhang, Sara Shayegi-Nik, Jessica Ly, Shaughna Cooper, Jason Trigg, Sophia Bartlett, Rolando Barrios, Julio S. G. Montaner, Kate Salters

**Affiliations:** 1 British Columbia Centre for Excellence in HIV/AIDS, Vancouver, Canada; 2 Experimental Medicine Program, Department of Medicine, University of British Columbia, Vancouver, Canada; 3 School of Population and Public Health, University of British Columbia, Vancouver, Canada; 4 British Columbia Centre for Disease Control, Vancouver, Canada; 5 Faculty of Health Sciences, Simon Fraser University, Burnaby, Canada; University of New Mexico Health Sciences Center, UNITED STATES

## Abstract

**Background:**

Hepatitis C virus (HCV) education may be changing following the simplification of HCV treatment and emergence of direct acting antiviral (DAA). We aimed to characterize HCV knowledge among people who recently completed DAA therapy.

**Methods:**

The Per-SVR (Preservation of Sustained Virologic Response) is a prospective cohort of patients who achieved a sustained virologic response upon successful completion of DAA therapy. The per-SVR study provided the sampling frame of participants who completed a psychometrically validated 19-item HCV knowledge scale at cohort entry (n = 227). To score the questionnaire, for each correct response one point was awarded, with no point for incorrect response. We assessed mean HCV knowledge score in the overall sample and mutually exclusive populations: people who inject drug (PWID) (n = 71); people with co-occurring HIV (n = 23); PWID and co-occurring HIV (n = 29), and others (n = 104) Using a latent class analysis based on distal outcome, we identified unobserved subgroups and assessed HCV knowledge amongst them.

**Results:**

Total mean (SD) percent of correct responses were 83 (11) in the overall sample; 83 (10) in PWID; 79 (12) in people with co-occurring HIV; 81 (10) in PWID and co-occurring HIV, and 84 (11) in rest of the sample Three latent groups were identified: baby boomers who ever experienced homelessness (n = 126); women sex workers who ever experienced homelessness (n = 68); men who inject drug, ever experienced homelessness and had ever diagnosis of mental health disorders (n = 18). Mean percent of correct responses were 85 (8), 82 (11), 85 (10), in latent class 1, 2, and 3, respectively.

**Conclusion:**

Patients successfully treated with DAAs had a high HCV knowledge. High knowledge and awareness of reinfection among complex patient groups often facing barriers to HCV care is encouraging and emphasizes the positive outcomes of universal access to treatment.

## Introduction

Hepatitis C virus (HCV) is a blood borne infection and one of the leading causes of end-stage liver disease [1]. In 2019, the World Health Organization (WHO) estimated that globally, 58 million people are chronically infected with HCV and 290,000 die annually from HCV-related liver diseases [2, 3]. HCV affects 250,000 Canadians, of which over 40% are unaware of their infection [4].

In North America, baby boomers (people born in 1945–1965) are a key population within the HCV epidemic [5, 6]. In British Columbia, Canada, it is estimated that 66% of people living with HCV are baby boomers [7]. However, a new US study found that the number of millennials (people born in 1981–1996) diagnosed with HCV is increasing and was comparatively equal to that of the baby boomers in 2018 [8]. The rapid increase in HCV infection among young adults coincides with the opioid crisis [9], which reportedly has led to a transition in HCV epidemic [10, 11].

With the advent of generally well-tolerated direct-acting antivirals (DAAs), HCV is now widely viewed as a curable disease, with a cure rate of 95% [12]. Despite this milestone, treatment initiation and inequality in care delivery remain the largest unaddressed gaps in HCV care [12]. A recent meta-analysis, found that DAA treatment uptake was 50% in Canada and 29% in the US [13]. Furthermore, there are disparities in HCV treatment uptake among marginalized populations due to comorbid conditions and concerns regarding reinfection, tolerability, adherence, and consequently potential for reduced virologic response [14, 15]. For instance, among people who inject drug (PWID), DAA treatment uptake is reported to be 40% in Canada, and 13% in the US [13].

A large body of research has investigated HCV knowledge. The majority of these data, however, are prior to treatment initiation and care engagement, or reflect pre-DAA era. The simplification of treatments may be significant for HCV knowledge as care is increasingly shifted to primary and community care settings and linkage to speciality care may be declining [16]. Therefore, patients’ education regarding HCV, including awareness of reinfection risk may be changing owing to reduced interactions with specialty health care providers or decreased health care exposure due to barriers complicating linkage to care in the DAA era, particularly among marginalized populations and people at higher risk of HCV reinfection [16–18].

In consideration of these factors, this study endeavors to describe patient characteristics and HCV knowledge in a cohort of people who successfully completed DAA-based HCV therapy and achieved undetectable viral load at the end-of-treatment.

## Methods

### Study design

The Per-SVR (Preservation of Sustained Virologic Response–pronounced “persevere”) is an ongoing study of patients who have successfully completed DAA-based therapy and achieved a sustained virological response (SVR) at the end-of-treatment in British Columbia (BC), Canada. Participants are eligible to join the study if they have completed HCV treatment and achieved SVR within three months of the study enrollment. Additional eligibility criteria include being ≥19 years of age, ability to communicate in English, and residing in BC. Eligible participants are monitored for HCV re-infection, as well as treatment and health-related outcomes, for a minimum of one year and maximum of four years after completion of DAA-based HCV therapy. They complete a total of ten visits for the duration of the study, are followed every three months in year one, and every six months in years two-four. Participants are required to complete study consent forms that detail their voluntary participation in the study. Each participant receives a $5 (CAD) honorarium for their initial screening and informed consent visit, and then a $40 (CAD) honorarium or equivalent gift card for all consecutive visits.

### Recruitment and data collection

Recruitment began in April 2017 and is still ongoing. Eligible participants are identified through associated hospitals, walk-in clinics, drop-in centers, community organizations, supervised injection facilities, and targeted outreach. Social media tools are used to recruit participants, including but not limited to Facebook, Grindr, and Twitter. We expect to recruit a total of 730 participants. The target sample size was calculated based on prior research conducted on HCV reinfection rates among different populations at high or low risk of reinfection; as of March 2021, 228 eligible participants have been recruited. Details on target sample size, recruitment process, and recruitment centres are provided in S1 File (S1 Appendix, S1 Table). Data on sociodemographic, substance use behavior, harm reduction services, health care engagement and other validated psychosocial metrics, are collected using standardized quantitative or semi-quantitative interviews conducted by skilled interviewers. Blood and urine samples are collected by clinical research associates. Presently, interviews are conducted either in-person or over the phone and samples are collected in-person at the nearest partnering laboratory location.

### Study sample

In the present study, we used a sample of per-SVR participants who completed a standardized HCV knowledge questionnaire at cohort entry as of 1 March, 2021 (n = 227). Only one participant had missing information on HCV knowledge questionnaire and therefore, excluded from the sample.

### Outcome of interest

The outcome of interest in the present study was mean percent of HCV knowledge score at cohort entry. We used a psychometrically validated and easy to use self-report measure of HCV knowledge scale. The scale reliability has been previously evaluated in terms of internal consistency based on coefficient alpha and test-retest reliability. The psychometric details of HCV knowledge scale is described elsewhere [19]. The standardized questionnaire is comprised of 19 items assessing general knowledge about HCV risk factors, modes of transmission and treatment options. Overall knowledge was assessed based on all the items (scale items #1–19) in the questionnaire. Specific knowledge domains (transmission, vaccination, and reinfection) were assessed based on regrouping of the related questions for each specific domain: transmission questions (scale items #1, #4, #7, #9, #11–14, #16, #18–19): vaccine-related questions (scale items #5–6): reinfection questions (scale items #15, #17) (Table 4). Scoring was calculated based on percent of correct responses; one point was awarded for each correct response (true), whereas no point was awarded for each incorrect (false) response. Knowledge was assessed within the overall sample, and among key populations of interest. Using a latent class method, we further identified latent groups in our sample and examined mean percent of HCV knowledge scores among them.

### Key populations

We created four mutually exclusive key groups to assess HCV knowledge among populations at higher risk of HCV infection. People who inject drug (PWID) who reported at least one time drug injection within a year prior to the study (n = 71); people with HIV coinfection, i.e. who had indication of positive HIV blood test at cohort entry (n = 23); people with indication of both injection drug use and HIV coinfection (n = 29); people with neither indication of injection drug use, nor HIV coinfection (n = 104).

### Explanatory variables

For the purpose of this study, explanatory variables were assessed at cohort entry based on self-reported demographic characteristics (age (median age and age categories ≤30, 31–49, 50–64, ≥65), gender (men, women [inclusive of cis- and transgender]), ethnicity (Caucasian, First Nations, Asian Decent, others), education (some school/post secondary, trade school/college, university), relationship status, employment (based on income), homelessness status); substance use (i.e. alcohol use, injection and non-injection drug use, cigarette/tobacco use); mental health disorders; HCV risk reduction practices (i.e. use of harm reduction services or practices, and patterns of sexual behavior); health care utilization. In addition to self-reported questionnaires, we used psychometrically validated scales at cohort entry for alcohol and drug use assessments. Specifically, we used the Alcohol Use Disorders Identification Test (AUDIT) which was developed by the World Health Organization as a simple method of screening for excessive drinking [20]; and the 10-item Drug Abuse Screening Test (DAST-10) which is a tool assessing drug use (not including alcohol or tobacco) in the past 12 months [21]; with scores above 6 categorized as high DAST score [22].To augment the self-reported data related to substance use, we further used urine screening samples obtained in the last three months. Laboratory data (i.e. data related to HIV and hepatitis B virus (HBV), and liver function) were assessed based on blood samples taken at cohort entry.

### Statistical analysis

Descriptive statistics (i.e. mean, median, standard deviation, and proportions) were run on our analytic sample. We used Kruskal Wallis test to compare HCV knowledge among four mutually exclusive key groups (i.e. PWID, HIV coinfection, PWID & HIV coinfection, rest of the sample). Chi-square/Fisher exact tests (for variables with two categories) or Kruskal-Wallis one-way analysis of variance (for variables with >2 categories) were used to compare HCV knowledge among key variables known to be associated with HCV knowledge.

We used latent class analysis (LCA) with distal outcomes to assess if HCV knowledge varies among unobserved (latent) subgroups within the per-SVR population. LCA with distal outcomes is a flexible approach to predict heterogeneity of a distal outcome (i.e. HCV knowledge score) from membership in latent classes, using multiple observed indicators [23–25]. Following observed variables in the study known to be associated with HCV knowledge outcomes were selected a priori, and incorporated in the model in a discrete categorical fashion: indicator of baby boomer birth cohort; gender (men, women [inclusive of cis and transgender],); ethnicity (Caucasian, First Nation, Asian descent, others); indication of injection drug use (last 12 months); history of sex work (ever); indication of homelessness (ever); mental health diagnosis (ever); HIV diagnosis (at cohort entry) [24]. First, we assigned individuals to latent classes based on posterior probability and theoretical interpretability. Second, we used Kruskal Wallis test to compare HCV knowledge among identified latent classes.

We first estimated a two-class model and then added one class at a time until we identified the model with the best fit. We used two main criteria to select the final LCA model: 1) epidemiological interpretability and meaningfulness; 2) multiple fit statistics based on information criteria, mainly relying on Bayesian Information Criteria (BIC) and Akaike Information Criteria (AIC), with lower values indicating better model fit. Additionally, we examined the quality of classification using two diagnostic statistics, i.e. entropy above 0.8 and the minimum average posterior probability of 0.70 [26]. Our final selected model was a 3-class model with a high entropy (0.94) and a similar or lower AIC/BIC to models with other classes (i.e. two-class, four-class, and five-class model).

A total of 13 people were not included in the LCA. We had missing data on gender (n = 1), ethnicity (n = 1), HIV test results (n = 2), and diagnosis of mental disorder (n = 2). For variable HIV test results, seven people did not have a HIV test and therefore were not applicable. LCA handles incompletely observed indicators using full information maximum likelihood technique under the assumption that data are missing at random [23]. The LCA approach is described in more detail in S1 File (S2 Appendix).

All analyses were performed using SAS programming software version 9.4 (SAS Institute Inc., Cary, NC, USA).

## Results

As of 1 March 2021, total number of 227 eligible participants completed the HCV knowledge scale at cohort entry and were included in the study. Baseline characteristics are described in Table 1. The median age (Q1, Q3) was 52 years (44, 59) and 64.8% (n = 147) were men. Baby boomers comprised 78.4% (n = 178) of the sample.

**Table 1:** Cohort profile of per-SVR participants who completed HCV knowledge questionnaire at cohort entry (n = 227) as of 1 March 2021.

**Distribution of key populations (mutually exclusive)**	
PWID, last twelve months	71 (31.2)
HIV Coinfection	23 (10.1)
PWID & HIV Coinfection	29 (12.7)
Neither PWID nor HIV Coinfection	104 (45.8)
**Demographics, mental health & laboratory data**	
**Gender**	
Men (cis and trans gender)	147 (64.7)
Women (cis and trans gender)	75 (33.0)
Age (years), median (Q1,Q3)	52 (44,59)
**Ethnicity**	
Caucasian	146 (64)
First Nations	24 (10.5)
Asian Decent	10 (4.4)
Other	46 (20.2)
Education (some school/post-secondary)	154 (67.8)
Relationship Status (Single)	127 (56.0)
**Living space**	
Room in hotel shelter/hostel	79 (34.8)
Apartment/condo	77 (33.9)
House student residence	43 (18.9)
Treatment/recovery house (for alcohol/drug use)	15 (6.6)
**Employment**	
Annual income (<50 K)	167 (73.5)
Income source (PWD)	93 (40.9)
**Homelessness, yes**	
Ever	172 (75.7)
Last three months	23 (13.3)
**Sex work, yes** ^#^	
Ever	74 (32.6)
Last three months	6 (2.6)
**HBSAG**	
Negative	158 (69.6)
Positive	<5
Not done	51 (22.4)
**Sexually transmitted infection, yes**	
Ever	116 (51.1)
Last three months	<5
**Mental health disorders, ever**	
No	108 (47.5)
Yes	117 (51.4)
**Types of mental health disorders**	
Depression	71 (60.6)
Anxiety	43 (36.7)
Trauma	36 (30.7)
Bipolar Disorder	21 (17.9)
Schizophrenia	16 (13.6)
ADHD	14 (11.9)
Personality Disorder	10 (8.5)
OCD	6 (5.1)
Other	6 (5.1)
**Health care utilization, yes**	
Regular primary health care	182 (80.1)
Accessing any health care, last three months	204 (89.8)
Seen specialist, last three months	101 (44.4)
**Substance use Profile**	
**Self-report screening scales, last 12 months**	
AUDIT scale, median (Q1, Q3)	3 (1, 7)
DAST scale, median (Q1, Q3)	5 (1, 7)
DAST scale > 6 ^##^	83 (36.5)
**Tobacco**	
Ever	212 (93.3)
Last three months	170 (74.5)
**Self-report injection drug use, yes**	
Ever	191 (84.1)
Last twelve months	100 (52.3)
Last three months	86 (37.5)
Public Injection, last three months	20 (23.8)
**Type of injection, ever**	
Cocaine	157 (69.1)
Heroin	153 (67.4)
Crystal	107 (47.1)
SpeedBall	82 (36.1)
Opiates	58 (25.5)
Goofball	55 (24.2)
Fentanyl	53 (23.3)
Crack Cocaine	48 (21.1)
Other	16 (7.0)
**Self-report injection at supervised facilities, yes**	
Ever	113 (59.1)
Last three months	47 (55.9)
**Frequency of fixing at supervised facilities, last three months**	
<25% of the time	32 (68.0)
>25% of the time	15 (31.9)
**Self-report non-injection drug use, yes**	
Ever	218 (96.0)
Last three months	147 (63.9)
**Type of non-injection drug, ever**	
Marijuana	202 (88.9)
Cocaine	182 (80.1)
Crack Cocaine	167 (73.5)
Mushrooms	151 (66.5)
LSD	143 (63.0)
Crystal	129 (56.8)
Heroin	128 (56.3)
Opiates	91 (40.0)
Ecstasy	91 (40.0)
Sleeping Pills	69 (30.0)
Benzo	68 (29.9)
Fentanyl	55 (24.2)
Ketamine (Special K)	43 (18.9)
Poppers	30 (13.2)
Other	25 (11.0)
**Urine Drug Screening, Last three months**	
People with at least one positive drug screen	164 (72.2)
**Type, %**	
Marijuana	85 (37.4)
Methadone	68 (29.9)
EDDP	67 (29.5)
Cocaine	64 (28.1)
Amphetamine	57 (25.1)
Morphine	55 (24.2)
Fentanyl	53 (23.3)
Ecstasy	25 (11.0)
Benzos	25 (11.1)
Buprenorphine**	10–15 (<7)
Oxycodone	<5

**Note:** Unless specified, variables are described in proportions number (%). Frequencies <5 are not reported for privacy reasons.

# Sex work refers to both genders, men and women.

**##:** DAST scale was categorized to a binary variable, high score (>6) versus low score.

** Buprenorphine value was masked to conceal oxycodone frequency for privacy reason.

Abbreviations: per-SVR: preservation of sustained virologic response; PWID: people who inject drugs; HCV: hepatitis C virus; HIV: human immunodeficiency virus; HBSAG: hepatitis B surface antigen; PWD: persons with disability; OCD: obsessive compulsive disorder; ADHD: attention deficit hyperactive disorder; AUDIT: alcohol use disorder identification test; DAST: drug abuse screening test; LSD: lysergic acid diethylamide; Benzo: benzodiazepine; EDDP: 2-ethylidene-1, 5-dimethyl-3, 3-diphenylpyrrolidine

History of experiencing ever homelessness was observed in 75.8% (n = 172) of the participants. Approximately half of the participants had reported ever being diagnosed with a sexually transmitted infection (51.1%, n = 116) or mental health disorder (51.4%, n = 117). Notably, self-reported depression was the most common mental health disorder (60.6%, n = 71). About 90% (n = 204) of participants reported regular access to any health care services in the last three months. A full 93.3% (n = 212) of participants had ever smoked tobacco/cigarettes, and 74.5% (n = 170) reported tobacco use in the last three months. In the last three months, injection drug use was reported in 37.5% (n = 86) and non-injection drug use was reported by 63.9% (n = 147) of the participants. Injecting at supervised injection facilities was reported among 55.9% (n = 47) of people who were using drugs in the last three months. Among people reporting substance use, Cocaine (69.1%, n = 157) and marijuana (88.9%, n = 202) were the most commonly reported injection and non-injection used substances, respectively, in the last three months.

We observed a high level of HCV knowledge in overall sample and among key population (Table 2*)*. Total mean (SD) percent of correct responses in the overall sample was 83 (11). In all the samples, knowledge of re-infection was slightly higher as compared to knowledge of transmission and vaccination. Interestingly, PWID had knowledge scores similar to that of overall sample, whereas people with HIV co-infection had slightly lower scores.

**Table 2:** Mean percent of correct responses to HCV knowledge scale within the overall sample and among the key populations.

HCV Knowledge	Overall sample (n = 227)	PWID** (n = 71)	HIV Coinfection (n = 23)	PWID & HIV Coinfection (n = 29)	Others (n = 104)	p-value
Total mean % of correct answers (SD)	83 (11)	83 (10)	79 (12)	81 (10)	84 (11)	0.10
Mean % of correct answers to transmission questions (SD)	87 (11)	88 (10)	83 (12)	85 (13)	88 (12)	0.27
Mean % of correct answers to vaccination questions, (SD)	77 (29)	78 (28)	72 (33)	78 (29)	78 (29)	0.86
Mean % of correct answers to re-infection questions (SD)	91 (21)	92 (19)	89 (21)	90 (25)	90 (21)	0.96

**Note:** Variables are mutually exclusive; p-values were derived from Kruskal-Wallis test.

** PWID refers to people who injected drugs in the last twelve months

Abbreviations: HCV: hepatitis C virus; PWID: people who inject drugs; HIV: human immunodeficiency virus; SD: standard deviation

No significant difference in knowledge was observed with respect to age (p = 0.28), gender (p = 0.83), treatment location (p = 0.61), indication of injection drug use in the past twelve months (p = 0.44), ethnicity (p = 0.51), ever experience of sex work (p = 0.08) or homelessness (p = 0.6). Diagnosis of mental disorder (p = 0.003) and higher level of education were positively associated with HCV knowledge (p = 0.01) (Table 3).

**Table 3:** Mean percent of correct responses to the HCV knowledge scale by key variables known to be associated with HCV knowledge.

	Total Mean % of Correct Answers mean % (± SD)	P-value
**Age**		0.28
≤30 (n = 6)	82 (14)	
31–49 (n = 77)	85 (8)	
50–64 (n = 125)	82 (12)	
≥65 (n = 19)	83 (11)	
**Gender (trans-&cisgender)**		0.83
Women (n = 75)	83 (10)	
Male (n = 147)	83 (11)	
**Treatment Location**		0.61
Community-based (n = 128)	83 (11)	
Hospital-based (n = 95)	83 (10)	
**Injection drug use, last twelve months**		0.44
No (n = 126)	83 (11)	
Yes (n = 100)	83 (10)	
**Ethnicity**		0.51
Caucasian (n = 146)	84 (10)	
First Nations (n = 24)	82 (9)	
Asian Descent (n = 10)	83 (12)	
Others (n = 46)	81 (12)	
**Education**		**0.01**
Some school/post secondary (n = 155)	81 (11)	
GED/trade school/college (n = 54)	86 (7)	
University (n = 18)	86 (10)	
**Sex work, ever**		0.08
No (n = 153)	82 (11)	
Yes (n = 74)	85 (8)	
**Homelessness, ever**		0.68
No (n = 55)	82 (11)	
Yes (n = 172)	83 (11)	
**Mental health disorder**		**0.00**
No (n = 108)	81 (12)	
Yes (n = 117)	85 (9)	

**Note:** Community-based locations include Pender Clinic, Columbia Street Clinic, Portland Hotel Clinic, Heatley Community Health Centre, Raven Song, and Downtown Community Health Centre in Vancouver; Surrey Lookout in Greater Vancouver; Positive Health Nanaimo in Vancouver Island (*S1 Appendix in S1 File);* p-values were derived from Kruskal-Wallis test for variables with >2 categories, and from Chi-square/Fisher exact tests for variables with two categories.

Abbreviations: HCV: hepatitis C virus; SD: standard deviation.

Knowledge of each individual item in the HCV knowledge scale is presented in Table 4. The lowest percent of correct responses (24.2%) was when people were queried if “People with hepatitis C can safely take any herbal medicine”. The highest percent of correct responses (99.1%) was when people were queried if “Hepatitis C can be given by hugs or handshakes”.

**Table 4:** Percent of correct responses to items of hepatitis C knowledge scale.

Scale Items	Percent of Correct Responses Overall Sample (n = 227)
1. People with hepatitis C can safely share their toothbrushes and razors with other people. (F)	209 (92.1)
2. People with hepatitis C can safely take any herbal medicine. (F)	55 (24.2)
3. People living with hepatitis C can damage their liver when they drink alcohol. (T)	221 (97.3)
4. People who received a blood transfusion in Canada before 1991 may have been infected with hepatitis C. (T)	205 (90.3)
5. There exists a hepatitis C vaccine that can be used to prevent people from getting infected with the hepatitis C virus. (F)	142 (62.5)
6. It is a good idea for people living with hepatitis C to be vaccinated against hepatitis A and B. (T)	209 (92.1)
7. Studies show that more than 60% of people who inject street drugs with ‘used needles’ are infected with hepatitis C. (T)	202 (88.9)
8. People can live with hepatitis C for many years without knowing that they have been infected with the virus. (T)	222 (97.7)
9. There is some risk that hepatitis C can be given to someone by snorting cocaine with shared straws, rolled money, etc. (T)	149 (65.6)
10. Some treatments for hepatitis C, such as interferon, can cause depression as a side effect in some patients. (T)	168 (74.0)
11. Using ‘new’ (i.e. never used before) needles, syringes, and equipment reduces the risk of being infected with hepatitis C. (T)	219 (96.4)
12. Babies born to hepatitis C pregnant women can be infected with hepatitis C at birth. (T)	164 (72.2)
13. Hepatitis C can be given to someone during sexual intercourse. (T)	197 (86.7)
14. Coughing and sneezing can spread hepatitis C. (F)	183 (80.6)
15. Successful hepatitis C treatments can result in the hepatitis C virus being completely removed (or cleared) from one’s blood. (T)	207 (91.1)
16. The hepatitis C virus can be spread from shared kitchen cups, plates or utensils. (F)	175 (77.1)
17. Once someone’s hepatitis C virus has been completely treated and cleared, one cannot get re-infected with hepatitis C. (F)	204 (89.8)
18. People can get infected with hepatitis C from tattoos and body piercing. (T)	219 (96.4)
19. Hepatitis C can be given by hugs or handshakes. (F)	225 (99.1)

We identified three latent classes, highlighting complex populations in our sample: latent class 1: baby boomers who ever experienced homelessness (n = 126, 59.4%); latent class 2: women sex workers who ever experienced homelessness (n = 68, 32.1%); latent class 3: men with indication of injection drug use, who ever experienced homelessness and had ever diagnosis of mental health disorders (n = 18, 8.4%). No significant difference in overall HCV knowledge, knowledge of transmission, re-infection, and vaccination was observed between these latent groups (Table 5). The details of LCA output is presented in S1 File (S3 Appendix, S2.1-S2.4 Table). Latent classes were further compared with respect to key variables associated with HCV knowledge outcome (S1 File: S4 Appendix, S3 Table).

**Table 5:** Comparison of HCV knowledge between latent classes identified using LCA.

HCV Knowledge	Latent Class 1 (n = 126)	Latent Class 2 (n = 68)	Latent Class 3 (n = 18)	P-value
Total mean % of correct answers (SD)	82 (11)	85 (8)	85 (10)	0.26
Mean % of correct answers to transmission questions (SD)	86 (12)	90 (8)	90 (11)	0.07
Mean % of correct answers to vaccination questions, (SD)	79 (30)	76 (27)	0.83 (0.24)	0.38
Mean % of correct answers to re-infection questions (SD)	90 (22)	94 (16)	86 (23)	0.23

Latent Class 1: baby boomers who ever experienced homelessness

Latent Class 2: women sex workers who ever experienced homelessness

Latent Class 3: men with indication of injection drug use who ever experienced homelessness and had ever diagnosis of mental health disorders

**Note:** p-values were derived from Kruskal-Wallis test.

Abbreviations: HCV: hepatitis C virus; LCA: latent class analysis; SD: standard deviation.

## Discussion

Our results indicate a high level of HCV knowledge among a cohort of patients recently successfully treated with DAAs. We found high HCV knowledge among the entire sample, as well as within key populations. Using LCA, we identified three complex latent groups in our sample: baby boomers with ever experience of homelessness; women sex workers with ever experience of homelessness; and men with a history of injection drug use, who ever experienced homelessness and had ever diagnosis of a mental health disorder. No significant differences in HCV knowledge between these groups were observed.

Previous studies have documented lower HCV knowledge in populations at high risk of HCV infection, particularly among PWID [27]. In our analysis, we observed no significant difference in knowledge between PWID versus those with no history of drug injection, and the knowledge score in PWID was comparable to that of the overall sample. This possibly reflects high engagement of per-SVR participants in harm reduction series including use of supervised injection facilities (i.e. 55.9% were injecting at a supervised facility in the last three months). Muncan and colleagues suggest that in comparison to the traditional health care settings, harm reduction facilities are identified as a more acceptable and effective source for enhancing HCV knowledge and providing HCV care among PWID as they allow convenient access to care in a non-stigmatizing environment [28]. These data highlight the importance of integration of substance use care and HCV treatment to improve overall health and patient education.

Previously, higher knowledge has been associated with higher education, being Caucasian, being a woman, and being employed [29, 30]. Further, a significant association has been described between knowledge of viral infections and socioeconomic status [31]. We did observe positive association between higher education and HCV knowledge. Our analysis did not show any significant difference in knowledge based on gender, age, treatment location, ethnicity, or socioeconomic status as reflected by indicators of ever experience of sex work or homelessness or identified latent groups using LCA method. Interestingly, we also observed that knowledge was slightly higher among people with co-occurring mental disorders. We speculate that our positive results are a reflection of adequate health care exposure and the removal of health care barriers for marginalized population. This is demonstrated in the data that in the last three months about 90% of the participants reported they had access to any type of health care, and 44% were able to see a specialist. Our results are in line with another Canadian study conducted by Mah and colleagues in a same setting who also reported increasing HCV knowledge amongst PWID [29]. It is also possible that participants developed healthy coping skills by enhancing their HCV knowledge on factors associated with reinfection or transmission risk to maximize their recovery experience acquired through DAAs. Reported low frequencies of sexually transmitted infection (<5), low percent of engagement in sex work (2.6%), and low percent of public injection (23.8%) in the last three months, support our speculation. An Australian study assessing knowledge among indigenous Australians who underwent HCV treatment also reported high knowledge in different domains such as transmission and testing, which promoted healthy behaviors among this population since diagnosis and treatment initiation [32].

It is worth noting that while knowledge of transmission and re-infection were noticeably high in our sample, the least knowledge was observed with respect to safe consumption of herbal medicine. This is while an African study has reported high prevalence of herbal remedies among newly diagnosed patients with HCV [33]. Herbal products are also popular among indigenous people and have been used for centuries to treat illnesses [34]. Simultaneous consumption of highly effective DAA and herbal products may not be safe and may cause liver injury [34]. Overall, data on complementary and alternative medicine and their efficacy among people with HCV infection are limited or report mixed results. A review by Liu J., et al., reported no firm evidence of efficacy related to the use of any medicinal herbs for HCV infection [35].

We acknowledge the present study has limitations. First, self-report respondent bias adherent to self-report studies, such as social desirability bias is possible. To minimize this type of bias, we performed our data collection using trained and skillful interviewers, as well as using psychometrically validated scales. Second, The HCV knowledge scale we have used includes largely questions related to transmission and fewer questions related to reinfection and vaccination. Therefore, knowledge scores related to reinfection and vaccination may not be well representative. However, it is demonstrated that this scale has a high reliability and validity across patient and healthcare providers and is well suited to inform HCV educational interventions [19]. Third, we acknowledge that our results may be biased due to the problem of confounding by indication. Previous HCV treatments is found to be associated with higher HCV knowledge. Individuals with previous HCV treatments would have multiple HCV-specific health care encounters providing the opportunity to leverage their knowledge. Higher HCV knowledge is also found to be associated with increased willingness to initiate DAA treatment [29], therefore it is likely that our cohort captures populations of patients who had high level of HCV knowledge to begin with. Additionally, implicit selection bias by health care providers leading to offering treatment to patients with less complex comorbidities and potentially better suited for DAA therapy is possible. We did not capture perception of health care professionals toward patient characteristics as a part of per-SVR study. However, by applying LCA method we were able to classify individuals based on combination of co-occurring characteristics which may indicate those with greater socioeconomic disadvantage and higher health complexities, therefore prone to lower HCV knowledge. We observed the HCV knowledge level among them was still high and similar to that of the overall sample. This may signify the impact of equal and broad access to HCV care, as even individuals at greater socioeconomic disadvantage benefitted from HCV treatment access and attained the same level of HCV knowledge as other groups in the study. Fourth, we used a general and high-level psychometric to assess HCV knowledge that may had not been tailored enough to PWID. Ascertainment of HCV knowledge among PWID may require more rigorous psychometrically tested measures specific to this population. A 2018 study using newly developed HCV injection-risk knowledge scale among young opioid users in New York City found that mean percent of correct responses was 75% and knowledge levels were highest for those previously tested for HCV, those with HCV antibody-positive status, and those who had received harm reduction information in various settings [36], which are consistent with our findings. Fifth, The LCA technique used in our analysis handles the missing data at random. We acknowledge that missing information on some variables such as HIV test results may not be at random. An alternative could be using an advanced LCA with imputation method for missing data [37]. We did not use this technique because only a total of 13 people from the overall sample (n = 227) were excluded from the LCA, seven of which were not applicable to be included. Furthermore, overall number of people included in the LCA model (n = 214) is slightly lower than what is suggested in the literature (i.e. 300) [26]. However, smaller samples are found to be adequate with simpler models (fewer indicators and classes) and “well-separated” classes [38]. Our model is relatively simple with three number of classes and nine variables and only nine missing values. It is not also recommended to have class sizes with fewer than 50 cases and classes should not contain less than 5% of the sample [26]. However, these suggestions have been relaxed and a number of publications have included class sizes smaller than 5% or 50 cases [39]. Nevertheless, we acknowledge that only 18 people are identified in latent class three that may reduce power of HCV knowledge comparison among latent classes.

Our results might change as per-SVR cohort and treatment uptake expands. Therefore, future analyses focused on change in knowledge over time once the recruitment is completed should be of interest. Strengths of our study are those inherent to prospective cohorts and high quality data on capturing a rare exposure such as HCV and timely capture of covariates. Through our analysis, we were able to characterize complex groups of patients who were recently cured of HCV using DAA-based therapies. Our results have important implications informing future endeavours targeting educational interventions in the DAA era.

### Conclusion

We observed high levels of HCV knowledge and comprehension of HCV transmission and reinfection risks among a sample of patients recently successfully treated for HCV in the DAA era. The knowledge was similarly high among complex populations with concurrent conditions reflecting importance of equal health care exposure, universal access to treatment, and prominence of integrative care on patient education and creating opportunities to promote knowledge acquisition among a more complex patient population. High awareness of reinfection risk among a sample of complex clients with multiple considerations further encourages DAA prescribing practices among health care providers. Continued evaluation of HCV knowledge levels among those who complete DAA treatment will be important to ensure that on-going engagement in follow-up care for those requiring surveillance for hepatocellular carcinoma or prevention of HCV re-infection, is maintained.

## Supporting information

S1 File S1 Appendix: Per-SVR target sample size and recruitment centres; S1 Table: Per-SVR Recruitment Centres in BC; S2 Appendix: Latent Class Models; S3 Appendix: LCA Output; S2.1-S2.4 Table: LCA output-response categories; S4 Appendix: Key characteristics of latent classes; S3 Table: Comparison of key variables known to be associated with HCV knowledge among identified latent classes. (DOCX)
