# Supplementary material for: Knowledge of hepatitis C and awareness of reinfection risk among people who successfully completed direct acting antiviral therapy
Source: PLoS One. 2022 Mar 23;17(3):e0265811. doi: 10.1371/journal.pone.0265811 (PMC8942206; doi:10.1371/journal.pone.0265811)
Supplement: S1 File — (DOCX) [file pone.0265811.s001.docx]

**Appendix 1. Per-SVR target sample size and recruitment centres**

**Target Sample Size:** In the present study, we used preliminary data collected for the per-SVR study from April 1, 2017 up to March 1, 2021. Recruitment is still ongoing until we reach the 730-target goal for participants. We calculated the sample size based on the prior research conducted on HCV reinfection rates among different populations of interest. While previous empirical studies on HCV reinfection are limited, a recent meta-analysis indicated that pooled HCV recurrence was 1.85/1000 and 22.32/1000 person years for “low risk” and “high risk” patients infected with mono-HCV, respectively. The pooled HCV recurrence for patients with coinfected HIV/HCV was 32.02/1000 person years. A Poisson regression module was used based on these reinfection rates to calculate the final sample size.

As of March 1, 2021, we had screened 385 participants with only 228 eligible for participation across sites in the metro Vancouver area. Individuals were ineligible if they did not have Hepatitis C, were not currently on treatment, or completed treatment more than three months ago. Study operations, including recruitment, were put on hold with the onset of the COVID-19 pandemic as of March 2020. We did not resume study operations until August 2020 and were restricted to only engaging with enrolled participants via phone. Our recruitment efforts include outreach and education sessions, which were not allowed during this time. In addition, most of our clinic and community partners had diverted resources to support the clients that they served which consequently limited their capacity to support the study recruitment. Recruitment for Per-SVR has only resumed in the summer/fall 2021 as our capacity for sample collection was also limited due to COVID-19 restrictions. Our aim is to reach the 730 target in the next couple of years as we expand our reach within metro Vancouver and to other sites on Vancouver Island as well as Northern and Interior BC.

**Recruitment:** Research is being carried at distinct research locations in Vancouver, including: Downtown Eastside (DTES) study office at Saint Paul’s Hospital and Pacific Gastroenterology Associates Research Institute; Vancouver General Hospital; Vancouver Coastal Health Community Centres (i.e. Downtown Community Center, the Pender Community Centre, the Raven Song Community Centre, and the Three Bridges Community Centre);. We have additional recruitment sites on Vancouver Island, Positive Health Nanaimo and Positive Wellness North Island in Comox and Campbell River, BC.

**Supplementary Table 1. Per-SVR Recruitment Centres in BC**

| **Recruitment Centres** | |
| --- | --- |
| Pender  SPH-IDC  DCHC  Raven Song  Lair Center  Vancouver General Hospital  SPH-PGA  Vancouver Native Health  Cedar Project  VIDC  Heatley CHC  Positive Health-Nanaimo**^$$^**  Surrey Lookout**^$^**  Columbia Street Clinic  BC Womens Hospital and Health Centre  Dr. John Farley  Oak Tree Clinic  Portland Hotel Clinic | 58 (25.5)  29 (12.7)  27 (11.8)  19 (8.3)  17 (7.4)  16 (7.0)  9 (3.9)  9 (3.9)  8 (3.5)  6 (2.6)  <5  <5  <5  <5  <5  <5  <5  <5 |

**Note:** All variables are described in proportions number (%). Frequencies <5 are censored for privacy reasons.

**$:** specifies Greater Vancouver area clinic; **$$:** specifies Vancouver Island clinic; rest of the centres/hospitals are located in Vancouver.

Abbreviations: SPH-IDC: Saint Pauls’ hospital-immunodeficiency clinic; DCHC: Downtown community health center; SPH-PGA: Saint Paul’s hospital- pacific gastroenterology associates; VIDC: Vancouver infectious disease centre; CHC: Community health centre; per-SVR: Preservation of sustained virologic response; BC: British Columbia.

**Appendix 2.** **Latent Class Models**

**Types of LCA Models:** We can apply LCA using two different techniques: 1) LCA with covariates, and 2) LCA with distal outcomes. The overall objective of both approaches is to explain population heterogeneity by identification of latent classes. In method 1 (i.e. LCA with covariates), interest lies in which characteristics predict latent class membership. In this method, the predictor is already observed and we are using that observed covariate to predict latent classes. For example, if we were to use method 1, the interest would be applying different categories of HCV knowledge (e.g. low versus high score) to predict latent class membership for each category. We used LCA with distal outcomes. In this method, the objective is to predict a distal outcome from latent class membership, using multiple observed indicators. Therefore, we can say that method 2 is in opposite direction of method 1, in which the predictor (i.e. subgroup memberships) is latent and the outcome (i.e. HCV knowledge) is manifest. More precisely, we are interested in the conditional distribution of a distal outcome, given latent class variable. The second method is more complicated because the predictor (true subgroup membership) is unknown. One of the most straightforward approaches to estimate the effect of latent class predictors on distal outcome is to assign individuals to latent classes based on their maximum posterior probability and theoretical interpretability. Then class membership is treated as known and a subsequent outcome analysis is conducted, which is what we have done in our analysis.

**Conducting LCA:** The standard procedure for conducting LCA is to conduct a sequence of models. The procedure is as such that we usually start with one-class model and we continue to run models with one additional class at a time until the best model is identified. Typically, model quality improves with additional classes until an optimal solution is found, and then model quality begins to decline. For example, in our analysis we ran two-class to five-class LCA models (i.e. started with two-class model and added 3 additional classes at a time). We observed that three-class model performed better that the two-class model. We ran four- and five-class LCA to assess if they outperform the three-class LCA. According to a combination of theoretical and statistical selection criteria, the LCA model with three-class was the optimal model that best fit our data.

**Model Selection and Fit Statistics:** the criteria used to decide the number of classes in LCA analysis is continuously evolving. Although there is no consensus about the best criteria for comparing latent class solutions, there is some agreement that a multiple fit and diagnostic statistics in combination with theoretical interpretability (i.e. epidemiologically meaningful in the context of our study) should be considered. The most common fit statistic is Bayesian Information Criterion (BIC). This criterion rewards parsimony in models. Lower BIC indicate better fit. Other information criterion (IC) can also be examined, including the Akaike information criterion (AIC), sample-size adjusted Bayesian information criterion (SABIC), and consistent Akaike information criterion (CAIC). Lower ICs also indicate better fit. Likelihood ratio tests could also be used. These statistics provide a p-value, which indicates if one model is statistically better than another one.

**Diagnostic Statistics:** In additional to evaluating fit, we need to review quality of classification (i.e. classification diagnostics). Although diagnostic statistics are not used to select the final class model, they are important for consideration. Two popular diagnostic statistics are posterior probabilities and entropy. Posterior probabilities is the average probability of being assigned to a class for individuals. Entropy indicates how accurately model defines classes. Typically, a cut-off value of greater than 0.80 is suggested for entropy.

**Missing Data:** Simple LCA models handles missing data using likelihood based estimation, which is the method used in our analysis. For complex data structures with a lot of missing data, an advance LCA with multiple imputation method could be used. In this method, we can obtain imputed values via an LCA model via an iterative Bayesian method, which imputes the latent class membership vector and the missing data values iteratively. We did not use this technique because only a total of 13 people from the overall sample (n=227) were excluded from the LCA. We had missing data on gender (n=1), ethnicity (n=1), HIV test results (n=2), and diagnosis of mental disorder (n=2). For variable HIV test results, seven people did not have a HIV test and therefore were not applicable.

**Limitations:** Overall number of people included in the LCA model (n=214) is slightly lower than what is suggested in the literature (i.e. 300). However, smaller samples are found to be adequate with simpler models (fewer indicators and classes) and “well-separated” classes. Our model is relatively simple with 3 number of classes and 9 variables and only nine missing values. It is not also recommended to have class sizes with fewer than 50 cases and classes should not contain less than 5% of the sample. However, these suggestions have been relaxed and a number of publications have included class sizes smaller than 5% or 50 cases. Nevertheless, we acknowledge that only 18 people are identified in latent class three that may

**References:**

Weller, B. E., Bowen, N. K., & Faubert, S. J. (2020). Latent Class Analysis: A Guide to Best Practice. Journal of Black Psychology, 46(4), 287–311. <https://doi.org/10.1177/0095798420930932>

Larose C, Harel O, Kordas K, Dey DK. Latent Class Analysis of Incomplete Data via an Entropy-Based Criterion. Stat Methodol. 2016;32:107-121. doi:10.1016/j.stamet.2016.04.004

**Appendix 3. LCA Output**

**Supplementary Table 2.1** LCA Output – Response Category 1

| **Variables** | Class 1 | **Class 2** | **Class 3** |
| --- | --- | --- | --- |
| Baby boomers (no) | 0.0538 | 0.3810 | 0.7586 |
| Gender (men) | 0.7811 | 0.2951 | 1.0000 |
| Ethnicity (Caucasian) | 0.7051 | 0.7051 | 0.5309 |
| Injection drug use-12 months** (no) | 0.4378 | 0.4378 | 0.0000 |
| Ever sex work (no) | 1.0000 | 1.0000 | 0.6742 |
| Ever homelessness (no) | 0.2966 | 0.2966 | 0.1573 |
| HIV test (positive) | 0.2658 | 0.2658 | 0.0000 |
| Diagnosis of mental disorder (no) | 0.5863 | 0.5863 | 0.0000 |

**Supplementary Table 2.2** LCA Output – Response Category 2

| **Variables** | Class 1 | **Class 2** | **Class 3** |
| --- | --- | --- | --- |
| Baby boomers (yes) | 0.9462 | 0.6190 | 0.2414 |
| Gender (women) | 0.2189 | 0.7049 | 0.0000 |
| Ethnicity (First Nations) | 0.0856 | 0.1675 | 0.0451 |
| Injection drug use-12 months** (yes) | 0.3039 | 0.6469 | 0.7210 |
| Ever sex work (yes) | 0.0000 | 1.0000 | 0.3258 |
| Ever homelessness (yes) | 0.7034 | 0.9100 | 0.8427 |
| HIV test (negative) | 0.7342 | 0.7301 | 1.0000 |
| Diagnosis of mental disorder (yes) | 0.4137 | 0.6401 | 1.0000 |

**Supplementary Table 2.3** LCA Output – Response Category 3

| **Variables** | Class 1 | **Class 2** | **Class 3** |
| --- | --- | --- | --- |
| Ethnicity (Asian) | 0.0236 | 0.0151 | 0.2566 |

**Supplementary Table 2.4** LCA Output – Response Category 4

| **Variables** | Class 1 | **Class 2** | **Class 3** |
| --- | --- | --- | --- |
| Ethnicity (Other) | 0.1857 | 0.2550 | 0.1674 |

****** The variable has been assessed among those who had ever indication of injection drug use.

| **Fit Statistics** |  |
| --- | --- |
| Log-likelihood | -1193.38 |
| G-squared | 417.73 |
| AIC | 493.73 |
| BIC | 621.64 |
| CAIC | 659.64 |
| Adjusted BIC | 501.23 |
| Entropy | 0.95 |

**Appendix 4. Key characteristics of latent classes**

**Supplementary Table 3.** Comparison of key variables known to be associated with HCV knowledge among identified latent classes

|  | **Latent Class 1 (n=128)** | **Latent Class 2**  **(n=68)** | **Latent Class 3**  **(n=18)** | **P-value** |
| --- | --- | --- | --- | --- |
| **Age**  ≤30  31-49  50-64  ≥65 | 0 (0)  29 (22.6)  90 (70.3)  9 (7.0) | <5  39 (57.3)  23 (33.8)  <5 | <5  8 (44.4)  <5  <5 | **<0.001** |
| **Gender**  Women  Men  Non-binary/other | 25-30 (20-25)  100 (78.1)  <5 | 44 (64.7)  20-25 (30-35)  <5 | 0 (0)  100.0  0 (0) | **<0.001** |
| **Treatment Location**  Community-based | 67 (52.3) | 44 (64.7) | 12 (66.6) | 0.47 |
| **Injection drug use, last twelve months**  Yes | 39 (30.4) | 45 (66.1) | 12 (66.6) | **<0.001** |
| **Ethnicity**  Caucasian  First Nation  Asian  Others | 91 (71.0)  11 (8.5)  <5  20-25 (15-20) | 39 (57.3)  11 (16.1)  <5  15-20 (20-25) | 8 (44.4)  <5  <5  <5 | **0.002** |
| **Education**  Some school/post secondary  Trade school/college  University | 82 (64.0)  34 (26.5)  12 (9.3) | 53 (77.9)  10-15 (10-15)  <5 | 11 (61.1)  7 (38.8)  0 (0) | 0.10 |
| **Sex work, ever**  Yes | 0 (0) | 68 (100. 0) | <5 | **<0.001** |
| **Homelessness, ever**  Yes | 90 (70.3) | 62 (91.1) | 15 (83.3) | **0.003** |
| **Mental health disorder**  Yes | 53 (41.4) | 44 (64.7) | 18 (100.0) | **<0.001** |

**Note:** Variables are reported in percentages. To censor frequencies <5, we have masked categories of some variables; p-values are derived using Kruskal-Wallis test.
